# Fine selection of up-regulated genes during ovulation by *in vivo* induction of oocyte maturation and ovulation in zebrafish

**DOI:** 10.1186/s40851-017-0065-8

**Published:** 2017-02-28

**Authors:** Wanlada Klangnurak, Toshinobu Tokumoto

**Affiliations:** 1 Integrated Bioscience Section, Graduate School of Science and Technology, National University Corporation Shizuoka University, Ohya 836, Suruga-ku, Shizuoka 422-8529 Japan; 2 Department of Biology, Faculty of Science, National University Corporation Shizuoka University, Shizuoka, 422-8529 Japan

**Keywords:** Ovulation, Steroids, Oocyte maturation, Zebrafish

## Abstract

**Background:**

Two essential processes, oocyte maturation and ovulation, are independently induced, but proceed cooperatively as the final step in oogenesis before oocytes become fertilizable. Although these two processes are induced by the same maturation-inducing steroid, 17α, 20β-dihydroxy-4-pregnen-3-one (17, 20β-DHP), in zebrafish, it has been suggested that the receptor, and thus the signal transduction pathway is different for each process. Although much progress has been made in understanding the molecular mechanisms underlying the induction of oocyte maturation, the mechanisms for inducing ovulation remain under investigation. In the present study, *in vivo* induction techniques that permit the induction of oocyte maturation and ovulation in living zebrafish (*in vivo* assays) were used to select highly up-regulated genes (genes associated with ovulation). Using an *in vivo* assay, ovarian tissues that induced only oocyte maturation could be obtained. This made it possible for the first time to distinguish maturation-inducing genes from ovulation-inducing genes. Using a genome-wide microarray of zebrafish sequences, the gene expression levels were compared among an ethanol (EtOH)-treated group (non-activated group), a diethylstilbestrol (DES)- or testosterone (Tes)-treated group (maturation-induced group), and a 17, 20β-DHP-treated group (maturation- and ovulation-induced group). Ovulation-specific up-regulated genes were selected. The mRNA expression levels of the selected genes were measured by quantitative polymerase chain reaction (qPCR).

**Results:**

Among 34 genes identified, three that showed ovulation-specific increases were selected as candidates potentially associated with ovulation. The ovulation-specific up-regulation of three candidates, slc37a4a, zgc:65811 and zgc:92184 was confirmed by qPCR.

**Conclusion:**

Our *in vivo* assay provides a new approach to precisely select genes associated with ovulation.

## Background

Oocyte maturation in zebrafish can be induced by the maturation-inducing hormone (MIH) 17α, 20β-dihydroxy-4-pregnen-3-one (17, 20β-DHP) [1, 2]. 17, 20β-DHP binds to a membrane-bound progestin receptor (mPR) and acts via non-genomic pathways to induce oocyte maturation [3, 4]. By contrast, the signals for ovulation are activated by 17, 20β-DHP through a nuclear isoform of the progesterone receptor (nPR) [5]. Thus, it has been suggested that the actual pathways for inducing oocyte maturation and ovulation involve a coordinated process between the genomic and non-genomic pathways induced by 17, 20β-DHP [6]. In zebrafish, oocyte maturation can be induced within three hours and ovulation within four hours *in vivo* [7]. Although considerable progress has been made in understanding the mechanism of oocyte maturation, additional studies are needed to develop a clearer understanding of the mechanisms underlying ovulation.

The gene expression profile observed during ovarian development provides basic knowledge for understanding the fish reproductive system. The transcription profile of the fathead minnow (*Pimephales promelas*) ovary in different ovarian stages (atretic, previtellogenic, vitellogenic, and post-ovulatory follicles) has been described [8]. The authors of that study provided a list of genes that might shift their expression levels during several ovarian stages. A study has reported on the changes in the expression of steroidogenic enzymes, including steroidogenic acute regulatory protein (StAR), during the primary growth of follicles and on the effect of human chorionic gonadotrophin (hCG) in zebrafish [9]. Analysis of transcriptional levels in pre-ovulatory rainbow trout (*Oncorhynchus mykiss*) ovaries revealed a number of genes linked to maturational competence [10, 11]. It is well known that maturation and ovulation are overlapping events [12, 13]. Thus far, it has not been possible to distinguish the changes in gene expression for the induction of ovulation from those for the induction of oocyte maturation. Studies of the expression profile of genes associated with ovulation in the ovulated eggs and follicles may be perturbed by genes associated with oocyte maturation.

Previously, we established a procedure that enabled the preparation of ovarian tissue containing oocyte maturation-induced oocytes *in vivo*. Similarly, ovulation can be induced in live zebrafish [7]. This technique makes it possible to select the up-regulated genes that induce ovulation by comparing the gene expression between genes associated with oocyte maturation in matured oocytes and both genes associated with oocyte maturation and ovulation in ovulated eggs. Recently, the role of eicosanoids in ovulation and spawning was revealed via an *in vivo* bioassay [14].

In the present study, we used an *in vivo* bioassay to prepare matured and ovulated ovarian samples. Specifically, the up-regulated genes that induce ovulation were selected by microarray analysis. The mRNA abundance of highly up-regulated genes was confirmed by qPCR analysis. The results indicate that this procedure is practical and can be used to select genes associated with ovulation.

## Methods

### Experimental animals

Zebrafish were raised in a proper chamber with a recirculating water system maintained at 28.5 °C under a 14 h light:10 h dark cycle [15]; the fish were fed a diet of brine shrimp in the morning and fish feed pellets (Croma, Kobe, Japan) in the evening. Female fish were raised until they possessed full-grown immature oocytes and were then used in this experiment. All experiments were conducted in accordance with procedures approved by the Shizuoka University Animal Care Committee.

### Experimental design for collecting tissue

To investigate genes associated with ovulation, artificial inductions of maturation and ovulation were conducted by an *in vivo* bioassay following the method reported by Tokumoto *et al*. [7]. Briefly, female fish possessing full-grown immature oocytes were selected from a mixed group of 10–50 males and females that had been held in a 20 cm × 25 cm × 25 cm square acrylic case with the standard water system used in our aqualab. Females were transferred into a glass case containing 100 ml of water per fish. The fish were exposed to agents *in vivo* by adding each agent, at a 10,000-fold stock in EtOH (final concentration; 0.01% EtOH, 0.1 μM 17, 20β-DHP, 5 μM diethylstilbestrol (DES), 10 μM testosterone (Tes)), to water and were then incubated at 28.5 °C. More than five fish were used for each treatment. Female zebrafish were treated for three hours under these conditions. After incubation, the female zebrafish were killed by spinal severance followed by dissection. Ovarian samples were cut from the body cavity under sterile conditions. One side of the ovary was placed in a 1.5 ml micro tube and immediately frozen in liquid nitrogen to preserve the RNA until extraction. To assess the oocyte developmental stage, the other side of the ovary was placed in fresh zebrafish Ringer’s solution (116 mM NaCl, 2.9 mM KCl, 1.8 mM CaCl_2_, and 5 mM HEPES, pH 7.2) and observed using a stereomicroscope. The specimen showing the best ovarian status based on observation after collection in each treatment was selected for RNA sample preparation. Three replicates were separately prepared from three different batches (more than 20 individuals each) of zebrafish.

### RNA extraction and reverse transcription for microarray

The total RNA for microarray analysis was extracted from the ovarian tissue using ISOGEN (Nippon Gene, Tokyo, Japan) in accordance with the manufacturer’s protocol. ISOGEN is a phenol-based pre-made reagent for RNA extraction.

Complementary DNA was prepared from 500 ng of total RNA from each replicate, as described in the manual for Agilent Low RNA Input Linear Amplification kit (Agilent Technologies, Palo Alto, CA, USA). Double-stranded cDNA was synthesized using the kit, and Cy3-labeled cRNA was prepared by cDNA *in vitro* transcription in the presence of cyanine 3-CTP dyes. Fluorescently labeled RNA was then purified with Qiagen RNeasy spin columns in accordance with the manufacturer’s protocol (Qiagen, Hilden, Germany). After purification, the cRNA was stored at –80 °C until use.

### Microarray

cRNA was fragmented and used to hybridize to the zebrafish G2519F 4X44K microarray containing 43,803 sequences (Agilent Technologies, Palo Alto, CA, USA). Hybridization, washing, and scanning were performed in accordance with the manufacturer’s protocol. Microarrays were scanned on a DNA microarray scanner (Agilent Technologies, Palo Alto, CA, USA) at a resolution of 5 μm. Raw digitized expression values from each probe set were extracted using feature extraction software, and the features were flagged manually for poor quality. The microarray data were then analyzed using Gene Spring ver. 11.0 (Agilent Technologies, Palo Alto, CA, USA). Three sets of samples from 3 biological replicates (as described above in Experimental design for collecting tissue) were analyzed by three separate arrays.

### Gene Selection

The median signal of the samples treated with EtOH, DES, Tes and 17, 20β-DHP from the microarray analyses were analyzed by Subio Platform ver. 1.18.4667 (Subio Inc., Amami, Japan). Two types of analyses were performed to find the candidate genes: statistical selection and non-statistical selection. For statistical selection, two separate statistical analyses were performed. The 17, 20β-DHP-treated group was separately compared with the groups treated with DES, Tes and EtOH. Any genes exhibiting at least a 1.8-fold greater expression in the 17, 20β-DHP-treated group were selected by the Venn diagram analysis. In addition, analysis of variance (ANOVA) was performed across all groups by using the basic plug-in to find genes that were expressed at significantly different levels among the four treated groups. Finally, overlapping genes that were up-regulated in the 17, 20β-DHP-treated group and the significantly different genes from ANOVA were selected. For non-statistical selection, any genes showing a 10-fold greater expression in the 17, 20β-DHP-treated group compared with the EtOH-, DES- or Tes-treated groups were selected. A Venn diagram analysis was performed with these groups. Central overlapped genes were selected as candidates for genes associated with ovulation (Fig. 2).

### Quantitative RT-PCR

The mRNA abundance of the first 20 genes from the 33 genes selected (Table 1) and a gene from the non-statistical gene selection (Table 2) was assessed by qPCR to confirm their expression levels in the EtOH-, DES-, Tes- and 17, 20β-DHP-treated samples. Specific primers were designed via Primer 3 [16] (Table 3). The annealing temperature gradient was investigated for all primers to find the most appropriate temperature for qPCR amplification (data not shown). A widely used reference gene, elongation factor 1 alpha (*ef1α,* GenBank accession number L47669), was tested using the same sample set to validate the normalization procedure.

**Table 1:** Top 20 candidates for genes associated with ovulation selected by the statistical selection method. The fold changes among treated samples are shown

Gene name	Accession #	Description	Fold change relative to EtOH			Fold change relative to DES			Fold change relative to Tes			Fold change relative to 17, 20β-DHP		
			DES	Tes	17, 20β-DHP	EtOH	Tes	17, 20β-DHP	EtOH	DES	17, 20β-DHP	EtOH	DES	Tes
cyp11a1	NM_152953	Danio rerio cytochrome P450, subfamily XIA, polypeptide 1), mRNA	31.7	1.3	433.7	<0.01	<0.01	13.7	0.8	24.9	340.8	<0.01	0.1	<0.01
zgc:136308	NM_001045247	Danio rerio zgc:136308, mRNA	1.2	4.5	24.5	0.8	3.6	19.7	0.2	0.3	5.4	<0.01	0.1	0.2
efna1	NM_200783	Danio rerio ephrin A1, mRNA	1.7	5.5	16.5	0.6	3.2	9.6	0.2	0.3	3.0	0.1	0.1	0.3
slc37a4a	NM_214738	Danio rerio solute carrier family 37 (glucose-6-phosphate transporter), member 4a, mRNA	7.9	6.4	15.3	0.1	0.8	1.9	0.2	1.2	2.4	0.1	0.5	0.4
slc25a10	NM_201172	Danio rerio solute carrier family 25 (mitochondrial carrier; dicarboxylate transporter), member 10, mRNA]	2.5	1.7	12.1	0.4	0.7	4.8	0.6	1.5	7.1	0.1	0.2	0.1
spata18	NM_001018678	Danio rerio spermatogenesis associated 18 , mRNA	4.1	5.2	11.6	0.2	1.2	2.8	0.2	0.8	2.2	0.1	0.4	0.4
sox21a	NM_131286	Danio rerio SRY-box containing gene 21a , mRNA	3.1	3.5	7.9	0.3	1.1	2.6	0.3	0.9	2.3	0.1	0.4	0.4
api5	NM_199540	Danio rerio apoptosis inhibitor 5, mRNA	3.2	3.1	7.0	0.3	1.0	2.2	0.3	1.0	2.2	0.1	0.5	0.4
nup85	NM_001003625	Danio rerio nucleoporin 85	2.3	3.6	7.0	0.4	1.6	3.0	0.3	0.6	1.9	0.1	0.3	0.5
gbx2	NM_152964	Danio rerio gastrulation brain homeo box 2	1.4	2.3	6.9	0.7	1.7	5.0	0.4	0.6	3.0	0.1	0.2	0.3
zgc:65811	NM_200552	Danio rerio zgc:65811 , mRNA	2.7	2.8	6.4	0.4	1.0	2.4	0.4	1.0	2.3	0.2	0.4	0.4
cnn2	NM_213349	Danio rerio calponin 2 , mRNA	2.5	1.5	5.6	0.4	0.6	2.2	0.7	1.7	3.6	0.2	0.5	0.3
CK139976	CK139976	AGENCOURT_16876226 NCI_CGAP_ZEmb3 Danio rerio cDNA clone IMAGE:7059552 5', mRNA sequence]	1.4	1.4	5.4	0.7	1.0	3.9	0.7	1.0	3.7	0.2	0.3	0.3
bcl3	XM_688922	PREDICTED: Danio rerio B-cell CLL/lymphoma 3, mRNA	1.3	1.6	4.5	0.8	1.2	3.5	0.6	0.8	2.9	0.2	0.3	0.3
asic2	NM_214788	Danio rerio acid-sensing (proton-gated) ion channel 2, mRNA	2.2	2.1	4.4	0.4	0.9	2.0	0.5	1.1	2.1	0.2	0.5	0.5
zgc:56525	NM_200279	Danio rerio zgc:56525, mRNA	1.9	2.3	4.2	0.5	1.2	2.2	0.4	0.8	1.8	0.2	0.5	0.5
asic4b	NM_214786	Danio rerio acid-sensing (proton-gated) ion channel family member 4b, mRNA	0.8	1.7	4.2	1.3	2.2	5.5	0.6	0.4	2.5	0.2	0.2	0.4
rhbdl3	NM_001017556	Danio rerio rhomboid, veinlet-like 3 (Drosophila), mRNA [NM_001017556]	0.6	1.3	3.7	1.7	2.2	6.3	0.8	0.5	2.9	0.3	0.2	0.3
LOC10000 3798	XM_001343224	PREDICTED: Danio rerio hypothetical protein LOC100003798, mRNA.	1.1	0.5	3.1	0.9	0.4	2.9	2.2	2.4	6.8	0.3	0.3	0.1
nptna	NM_001160156	Danio rerio neuroplastin a, mRNA	1.5	1.6	3.1	0.7	1.1	2.1	0.6	0.9	1.9	0.3	0.5	0.5

**Table 2:** Two genes associated with ovulation selected by the non-statistical selection method. The fold changes among treated samples are shown

Gene name	Accession #	Description	Fold change relative to EtOH			Fold change relative to DES			Fold change relative to Tes			Fold change relative to 17, 20β-DHP		
			DES	Tes	17, 20β-DHP	EtOH	Tes	17, 20β-DHP	EtOH	DES	17, 20β-DHP	EtOH	DES	Tes
cyp11a1	NM_152953	Danio rerio cytochrome P450, family 11, subfamily A, polypeptide 1	31.7	1.3	433.7	0.0	0.0	13.7	0.8	24.9	340.8	<0.01	0.1	<0.01
Zgc:92184	NM_001002344	Danio rerio zgc:92184	1.4	0.9	20.7	0.7	0.6	14.9	1.1	1.6	23.6	<0.01	0.1	<0.01

**Table 3:** Primers used for qPCR analyses of candidate genes.

Target gene	Accession #	Forward Primer 5' to 3'	Reverse Primer 5' to 3'	product size, bp	Ta, °C
cyp11a1	NM_152953	AAAGCCTGAAGACGGTGCTA	AGCAGGACGCCATATTTTTG	117	60
zgc:136308	NM_001111227	GCAAACACGACACAACTCCTGC	TGTGTCCTCCATCAGGTCTGTTTAC	130	56
efna1	NM_200783	AGCAGTTGGCGAAGGTGATG	CGGTAATGGAGGAGGCGTTC	99	56
slc37a4a	NM_214738	CTCCAGCAAAAATGAAAGCA	CACCCCAAACACCACCAG	91	56
Slc25a10	NM_201172	TAATATACTCACACACTTCCTG	CTGTATTCTCCTTTAGAGTTC	114	56
Spata	NM_001018678	AGAAATAACACTCAAAGAGG	AGACTACAAGGAGAAACACT	174	55
sox21a	NM_131286	GTTCCCTCATCTTATGTACT	TTAAACTCCACTCATATCGT	92	56
api5	NM_199540	GAGTCAAATCTTACCTTTCA	ACATACATCAGGGCATAATA	86	-
nup85	NM_001003625	CACTCTTACAGACCATGCCCATATT	CCAGTGTCTCCATTTCACATCAAAC	80	60
gbx2	AF288762	CATTAACACAACCATAATCC	GTCACTAACACAGTCTCACAT	106	-
zgc65811	NM_200552	TCCTCATGTTAGTTTAAGGTCACGG	ACAGAAATGAAAGAGAAGCAGAAGT	90	60
cnn2	NM_213349	GGACTACAGATGGGAACAAATAAAT	ATGTGTGACTTGGGATAATACAGAT	92	60
CK139976	CK139976	CAGTATCTGCGATGTTTAATGTCAG	GCTGTCTGAGTCTTCCATTTGA	102	61
bcl3	XM_688922	TGAAGAAAGAGGTGTGAGTTGATAG	TTAAAGAGACACAATGCTGAACGAA	115	61
asic2	NM_214788	AATGTTCTTTGAGGATGGATGGTT	GTGACCTTGTATTTAGATTGAGAGC	80	60
zgc:56525	NM_200279	CAACATTTCCTCCAGTGCTAAAG	CTTCAGTTCACTCTGCATCTTATTC	94	55
asic4b	NM_214786	GAGGAGTACATCAGAGACAACTTTC	CTTTCTTCTGCTCAATCGTTTCATA	82	57
rhbdl3	NM_001017556	TCACTATAACAGAGGTTGTTGTCTT	TTCAGGAAGTATGGCGATGATAC	88	-
LOC100003798	XM_001343224.1	GTACCTCAGTCAATCTCTAATCCTC	GAACCTTTGTTCATCTCTTCTGTTT	85	60
nptna	NM_001160156	ATACAGGCATTTCCAGGCTTTATTT	AAAGAACTGTCCAACCAGAATCAT	110	60
zgc:92184	NM_001002344	CCCGTGGCGGCGATATGCTT	TCCCCCGCAGCGTCTGATGA	544	56
ptgs2a	NM_153657	ATGTTTGCTTTCTTCGCCCA	AGATCCACTCCATGACCCAG	101	55
ef1α	L47669	CTTCTCAGGCTGACTGTGC	CCGCTAGCATTACCCTCC	358	60

RNA from four treated samples was extracted from ovarian tissue, again using ISOGEN. Total RNA (1 μg) was reverse transcribed using illustra Ready-To-Go RT-PCR Beads (GE Healthcare Life Sciences, Buckinghamshire, UK), following the manufacturer’s instructions. The qPCR reactions were performed in a 20 μl volume that contained 5 μl of 10 times-diluted cDNA, 1 μl of each primer (10 μM), and 10 μl of SYBR green PCR Master Mix (Roche Applied Science, Mannheim, Germany). Real-time qPCR was conducted by LightCycler® Nano System (Roche Applied Science, Mannheim, Germany). The thermal cycle began with an initial denaturation step at 95 °C for 5 min, followed by 45 cycles of denaturation at 95 °C for 10 sec, annealing at Ta°C (Table 3) for 10 sec, and extension at 72 °C for 15 sec. The final melting curve analysis was observed at 65 °C for 20 sec, followed by 95 °C for 20 sec. The mRNA abundance of each target gene was calculated from a serially diluted pooled cDNA, and each sample was normalized against its reference gene expression level. Biological triplicates were observed for each treatment. Triplicate reactions were performed for each individual sample. The normalized mRNA abundance was calculated as the mean ± SE.

### Statistical analyses

The mRNA abundance of each gene among the different treated samples was computed using the Kruskal-Wallis one-way ANOVA nonparametric test because not all of the data set met the ANOVA assumptions. The Mann-Whitney *U* test was calculated to compare whether any significant difference existed between the treated sample pairs. Significance was set at *P* ≤ 0.05.

## Results

**Fig. 1:**
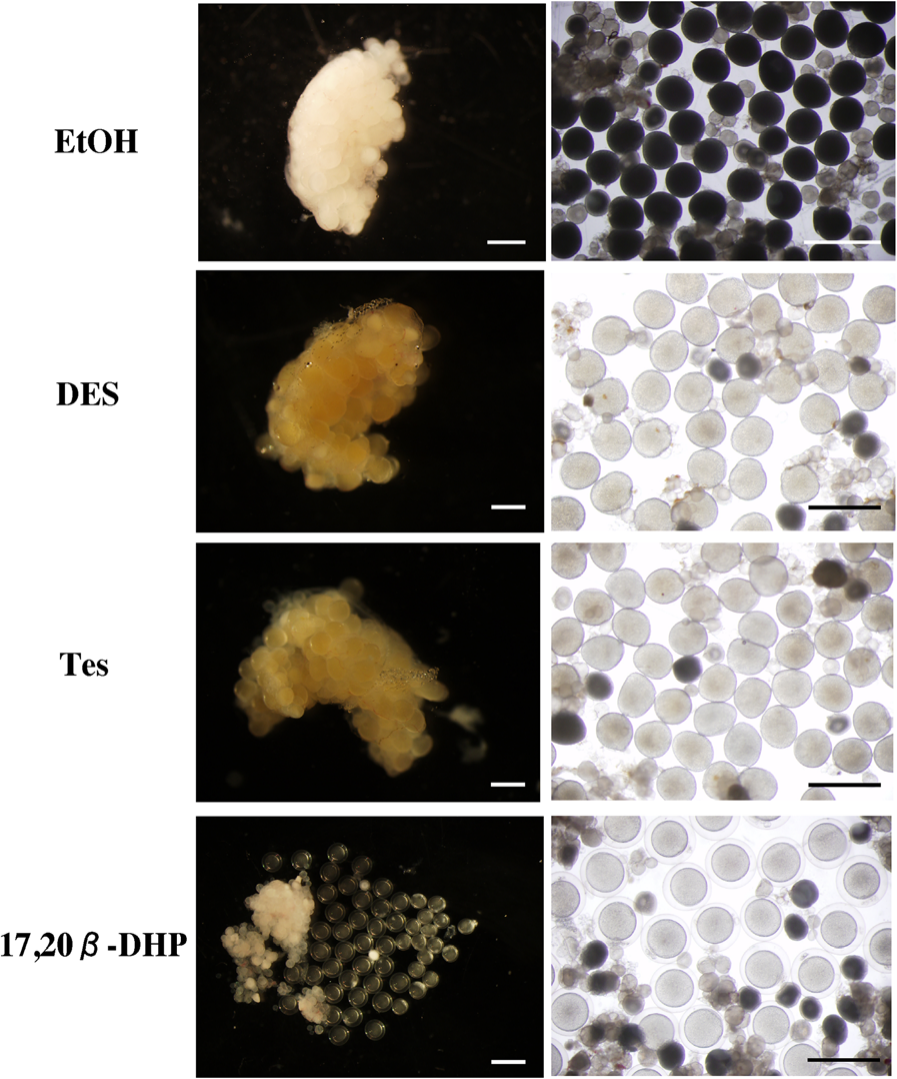
The *in vivo* bioassay was performed at a final concentration of 5 μM of DES, 1 μM of Tes or 0.01 μM of 17, 20β-DHP. One side of the ovary was observed by stereomicroscopy. The morphologies of the ovarian samples after three hours of treatment by EtOH, DES, Tes and 17, 20β-DHP were photographed. Ovaries before (*left panels*) and after (*right panels*) splitting are shown. After treatment with EtOH, the oocytes remained opaque and showed no morphological change after exposure to water. Oocytes after treatment with DES or Tes became transparent. A fertilization membrane developed in oocytes ovulated by 17, 20β-DHP treatment after exposure to water. Scale bars indicate 1 cm

As reported in 2011 by Tokumoto et al., oocyte maturation and ovulation can be induced by adding agents to the water in which the fish are maintained [7]. Using this *in vivo* bioassay method, we succeeded in preparing maturation-induced ovarian samples by DES or Tes treatment and ovulation-induced samples with 17, 20β-DHP treatment. Oocytes showed dramatic morphological changes in the samples treated with DES and Tes compared with the control treatment (EtOH) (Fig. 1). Oocyte maturation was induced within three hours after adding DES or Tes to the water, but ovulation did not progress. Oocytes underwent germinal vesicle breakdown and became transparent, which is a morphological characteristic of matured oocytes [7, 12]. In this manner, an ovarian sample that exhibited only oocyte maturation could be prepared *in vivo*.

17, 20β-DHP induced both oocyte maturation and ovulation, and fertilizable eggs were obtained at four hours. Oocytes in 17, 20β-DHP-treated fish expelled their follicles, and a fertilization membrane developed after contact with water, which is a morphological characteristic of ovulated eggs [7, 12]. We compared the mRNA expression levels in ovaries of fish treated with the following: EtOH (control group); DES or Tes (oocyte-maturation-induced group); or 17, 20β-DHP (an oocyte-maturation- and ovulation-induced group). To select the genes responsible for ovulation, we set the time period for preparing the mRNA at three hours after adding the agents to the water. Because we planned to analyze the changes in gene expression levels in the whole ovary, it was necessary to excise the whole ovary. To avoid the destruction of the ovary due to ovulation, the samples had to be obtained before ovulation. We speculated that the expression of genes associated with ovulation would have already started one hour before ovulation.

Transcriptome analysis was conducted using microarrays to select genes that were up-regulated during ovulation. We compared the expression levels of mRNA from ovaries in fish treated with EtOH, DES, Tes or 17, 20β-DHP using a conventional microarray containing probes of genome-wide variety (Agilent 4x44k). The signals from samples treated with 17, 20β-DHP, DES or Tes were significantly higher for nearly all of the genes than were the signals from samples treated with EtOH (data not shown). Although it was difficult to identify alterations in gene expression during the reproductive course, we identified the transcriptional levels of genes associated with ovulation by comparing signals between matured and ovulated oocytes.

**Fig. 2:**
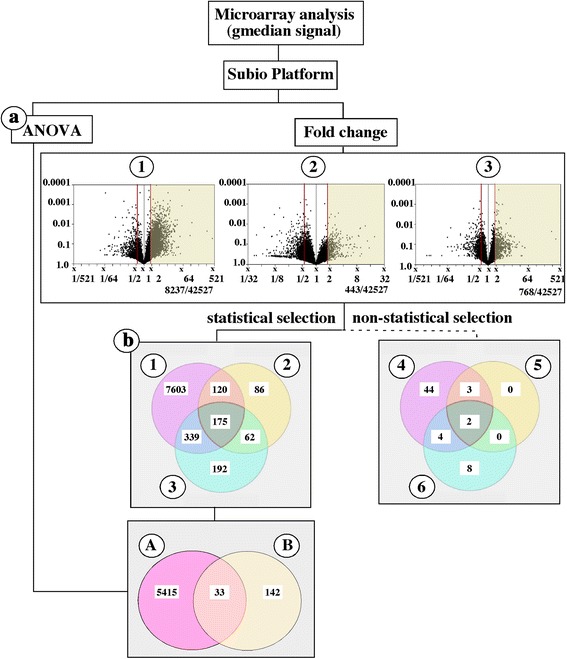
A diagram showing the process for selecting candidates of genes associated with ovulation by the Subio platform (Subio Inc., Amami, Japan). In standard statistical selection, ANOVA was performed across all groups to detect significant differences in both up- and down-regulation among all treated groups (*P* ≤ 0.05) (**a**). More than 1.8-fold greater expressions in 17, 20β-DHP than in EtOH (1) DES (2) or Tes (3) have been reported. Overlapping genes among these three groups were selected by Venn diagrams (**b**). Finally, overlapping genes in categories A and B were selected. Thirty-three genes were selected as candidates for genes associated with ovulation. Twenty highly elevated genes analyzed by qPCR were listed in Table 1. For non-statistical gene selection, more than 10-fold greater expressions in the 17, 20β-DHP-treated sample compared with the EtOH- (4) DES- (5) or Tes- (6) treated samples were analyzed by Venn diagrams. Two genes were selected as candidates for genes associated with ovulation (Table 2)

The genes that were up-regulated to induce oocyte maturation (genes associated with oocyte maturation) were excluded from the 17, 20β-DHP-treated group that contained both genes associated with maturation- and ovulation. Genes that were significantly up-regulated only in the 17, 20β-DHP treated group were selected. In the standard statistical selection, ANOVA was performed across all groups to detect significantly different up- and down-regulated genes among all treated groups (*P* ≤ 0.05) (Fig. 2). A total of 5448 genes were identified (category a in Fig. 2). Subsequently, genes were selected that represented expression levels that were up-regulated more than 1.8-fold in the 17, 20β-DHP-treated group. By comparing the EtOH-, DES- and Tes-treated groups, 8237 (1), 443 (2) and 768 (3) genes were selected, respectively. Overlapping genes among these three groups were selected by Venn diagram analysis (category b in Fig. 2). Finally, overlapping genes in categories A and B were selected. Thirty-three genes were selected as candidates for genes associated with ovulation (Table 1). For non-statistical gene selection, 53, 5 and 14 genes that were 10 times higher in 17, 20β-DHP compared with the EtOH- (4) DES- (5) and Tes- (6) treated groups, respectively, were analyzed by Venn diagram. Only two genes were selected as candidates for genes associated with ovulation by this method (Table 2).

**Fig. 3:**
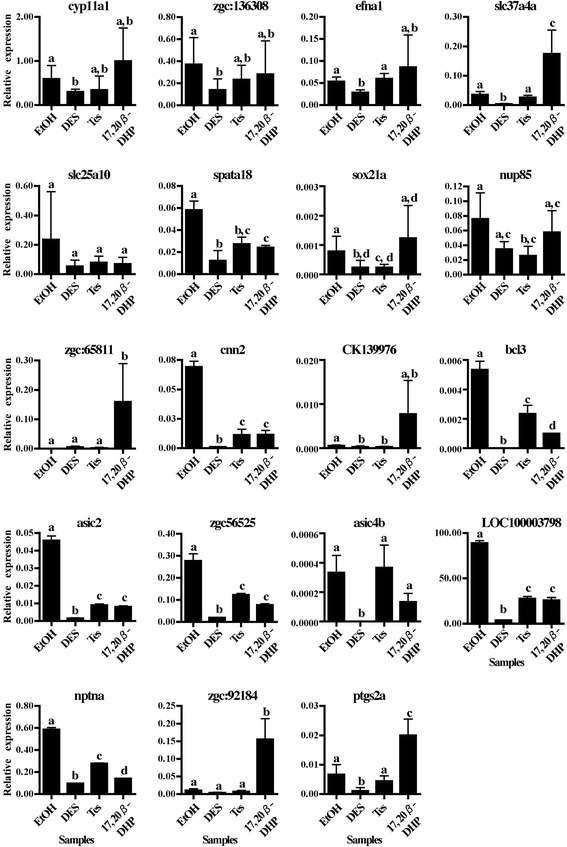
qPCR analysis of candidate genes associated with ovulation listed in Tables 1 and 2 with ptgs2a as a positive control was conducted using cDNAs prepared from ovaries from fish treated by EtOH, DES, Tes or 17, 20β-DHP for three hours. The mRNA abundance was observed in triplicate for each sample and all data were normalized with the number of transcripts of elongation factor 1α (EF1α) in each sample. Expression values are represented as the mean ± SE of three independent samples. Different letters represent significant differences among the data (*P* ≤ 0.05)

**Fig. 4:**
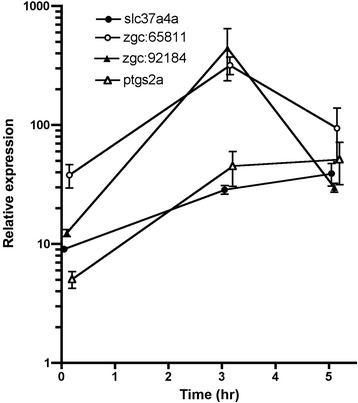
Total mRNAs were prepared from samples at 0, 3, 5 hour treated with 17, 20β-DHP. We conducted qPCR analysis of slc37a4a, zgc:65811, zgc:92184 and ptgs2a. The mRNA abundance was observed in triplicate for each sample and all data were normalized with the number of transcripts of elongation factor 1α (EF1α) in each sample. Expression values are represented as the mean ± SE three independent samples

The expression levels of the first 20 genes from the statistical selection and 1 gene from the non-statistical selection were analyzed by qPCR with ptgs2a, well-known up-regulated gene before ovulation, as a positive control [14]. Although 18 of them (with the exception of api5, gbx2 and rhbdl3) showed excellent amplification when detected by absolute quantification analysis and melting temperature profiles (data not shown), only three genes, slc37a4a, zgc:65811 and zgc:92184 showed significantly greater expression in the 17, 20β-DHP-treated group (Fig. 3, *P* < 0.05 with all other groups). An inconsistent expression trend was observed between the microarray detection and qPCR analysis in the other genes. As a result, we selected only three genes, slc37a4a, zgc:65811 and zgc:92184, as candidates potentially associated with ovulation by the microarray analysis platform. We then conducted time course analysis of expression levels of selected genes and ptgs2a during DHP treatment. As expected, levels of all four genes were elevated at three hours, but slc37a4a and ptgs2a were more strongly up-regulated at five hours (Fig. 4). This suggests zgc:65811 and zgc:92184 as the genes most likely to be associated with ovulation.

## Discussion

The *in vivo* bioassay established by Tokumoto et al. (2011) provided a novel means of distinguishing the pathways that induce ovulation and are known to be induced by genomic actions from the pathways involved in oocyte maturation, the non-genomic action-dependent pathways [7]. This *in vivo* assay is a practical technique for preparing matured and ovulated ovarian samples that was created to understand the influence of endocrine disrupting chemicals (EDCs) and steroid hormones on oocyte maturation and ovulation in live zebrafish. Both diethylstilbestrol (DES) and a naturally occurring steroid hormone, 17, 20β-DHP, were effective when these compounds were externally applied to a water column containing live fish. The results showed that fish kept in water containing 17, 20β-DHP induced both maturation and ovulation, whereas fish treated with DES underwent oocyte maturation and stopped at this stage. A high expression of genes associated with oocyte maturation was observed in mRNA extracted from matured oocytes. Similarly, both genes associated with maturation and ovulation are highly expressed in ovulated eggs. Genes that are highly expressed in ovulated eggs, but exhibit a low expression in matured oocytes could be selected as candidates for genes associated with ovulation.

The ovulation-inducing pathway has been studied for more than two decades [17], which has provided a deeper understanding of the reproductive system. Metalloproteinases responsible for the rupture of follicle cells were identified in Medaka [18]. The first transcriptome analysis was conducted in rainbow trout (*Oncorhynchus mykiss*) [11]. The gene sets that were up- or down-regulated during spawning were identified by a microarray analysis. Many biological activities, such as proteolysis, inflammation, coagulation, vasodilatation, and angiogenesis, have been reported as essential mechanisms in the acquisition of maturational competence and ovulation occurring in the pre-ovulatory ovarian follicle [19]. Recently, specifically up-regulated genes in the pre-ovulatory, peri-ovulatory, and peri-spawning intervals have been identified in zebrafish [14]. However, none of these studies eliminated the maturation-inducing genes that are expressed in ovulated ovarian tissue, although it is well known that oocyte maturation and ovulation are overlapping events [12, 13]. This current study is the first to eliminate maturation-inducing genes from ovulation-inducing genes.

The microarray analysis identified a number of up-regulated genes by the statistical selection method. Of 20 genes, only solute carrier family 37 (the glucose-6-phosphate transporter) member 4a (slc37a4a) and zgc:65811 were confirmed as specifically up-regulated during ovulation by qPCR analysis. The mRNA of slc37a4a was reported to be expressed ubiquitously in human [20], whereas its variant is primarily expressed in the brain, heart, and skeletal muscle [21]. This well-characterized protein is one of four protein families that play important roles as sugar transporters [22]. The slc37a4a is located on the endoplasmic reticulum (ER) via 10 transmembrane domains [23]. It encodes the glucose-6-phosphate transporter (G6PT) protein, which plays a central role in the translocation of glucose-6-phosphate (G6P) between the cytoplasm and the lumen of ER [24]. A mutation in the G6PT gene causes a deficiency in the transport of G6P into the lumen of the ER, which leads to type Ib glycogen storage disease [25]. This finding supports the idea that G6PT plays an important role in intracellular glucose homeostasis.

In dysregulated myocardial glucose metabolism, the intracellular accumulation of glucose 6-phosphate (G6P) can activate endoplasmic reticulum stress (ERS) [26]. It may be that high expression of slc37a4a (G6PT) results in excess G6P in the cell, thereby inducing ERS, leading to apoptosis which in turn induces ovulation [27]. Another candidate zgc:65811 was not annotated, but showed relatively high similarity with CD9 antigen-like gene of common carp (64%). CD9 antigen has previously been shown to be involved in fertilization in knockout mice [28]. Thus it can be speculated that elevation of expression of zgc:65811 before ovulation was to prepare for fertilization.

Another candidate for gene associated with ovulation, zgc:92184, is also not annotated. Blast analysis showed the highest similarity with the GTPase IMAP family member 7-like (LOC100005907) (Gimap7), with 82% identity. Based on this high sequence similarity, we speculate that the biological function of the unknown zgc:92184 gene may be analogous to that of Gimap7.

The Gimap7 gene corresponds to GTPase of the immunity-associated protein family (GIMAPs). GIMAPs are related to immunological functions, such as lymphocyte survival, thymocyte development and apoptosis regulation in cells of the mammalian immune system [29, 30]. It has been suggested that GIMAPs can play opposite roles to regulate the survival of lymphocyte cells; for example, GIMAP4 and GIMAP5 function differently in the regulation of apoptosis. GIMAP4-deficient rats are resistant to apoptosis [31], whereas GIMAP5-deficient mice showed higher rates of apoptosis [32]. Although the role of GIMAP7 in stimulating apoptosis is still uncertain, there are possibility that zgc:92184 in zebrafish is related to apoptosis. Apoptosis has been shown to be an essential mechanism for follicle rupture and leads to the completion of ovulation [33–35]. Two highly up-regulated genes found in this study might have the roles in induction of ovulation through the regulation of apoptosis.

Unfortunately, inconsistent expression levels between the microarray and qPCR analyses have been detected in many genes. The cross hybridization of mRNA resulting in an inaccurate detection of signals in the microarray analysis must be considered. This weakness can lead to the misinterpretation of the gene expression profile [36]. RNA sequencing, which is a modern approach to detect transcriptome profiling, should be adopted to obtain a precise quantity of transcripts and identify other ovulation-inducing genes. Naturally matured and ovulated samples need to be observed further to confirm the transcriptional levels of these genes.

The biological processes underlying the ovulation of other related genes reported in this study should be examined using the recently developed gene knockout technique. This study provides a new system for discovering genes that play essential roles in ovulation.

## Conclusion

The *in vivo* assay using living zebrafish allowed us to select genes that are specifically up regulated to induce ovulation. Candidates for genes associated with ovulation, slc37a4a, zgc:65811 and zgc:92184, represented high expression in 17, 20β-DHP-exposed samples (ovulated sample). Precise selection by *in vivo* assay provides new insights into the molecular mechanisms regulating ovulation.

## References

[1] Kondo T, Yanagawa T, Yoshida N, Yamashita M (1997). Introduction of cyclin B induces activation of the maturation-promoting factor and breakdown of germinal vesicle in growing zebrafish oocytes unresponsive to the maturation-inducing hormone. Dev Biol.

[2] Tokumoto T, Tokumoto M, Horiguchi R, Ishikawa K, Nagahama Y (2004). Diethylstilbestrol induces fish oocyte maturation. Proc Natl Acad Sci U S A.

[3] Zhu Y, Rice CD, Pang Y, Pace M, Thomas P (2003). Cloning, expression, and characterization of a membrane progestin receptor and evidence it is an intermediary in meiotic maturation of fish oocytes. Proc Natl Acad Sci U S A.

[4] Tokumoto M, Nagahama Y, Thomas P, Tokumoto T (2006). Cloning and identification of a membrane progestin receptor in goldfish ovaries and evidence it is an intermediary in oocyte meiotic maturation. Gen Comp Endocrinol.

[5] Zhu Y, Liu D, Shaner ZC, Chen S, Hong W, Stellwag EJ (2015). uclear progestin receptor (pgr) knockouts in zebrafish demonstrate role for pgr in ovulation but not in rapid non-genomic steroid mediated meiosis resumption. Front Endocrinol (Lausanne).

[6] Tokumoto T (2014). Zebrafish as a model for reproductive biology and environmental screening. In: Charles A, Lessman CA, Ethan CA, vol., editor. Zebrafish topics in reproductive toxicology and development.

[7] Tokumoto T, Yamaguchi T, Ii S, Tokumoto M (2011). In vivo induction of oocyte maturation and ovulation in zebrafish. PLoS One.

[8] Villeneuve DL, Garcia-Reyero N, Martinovic D, Cavallin JE, Mueller ND, Wehmas LC, Kahl MD, Linnum AL, Perkins EJ, Ankley GT (2010). Influence of ovarian stage on transcript profiles in fathead minnow (Pimephales promelas) ovary tissue. Aquat Toxicol.

[9] Ings JS, Van Der Kraak GJ (2006). Characterization of the mRNA expression of StAR and steroidogenic enzymes in zebrafish ovarian follicles. Mol Reprod Dev.

[10] Bobe J, Maugars G, Nguyen T, Jalabert B (2003). Specific gene expression profiles are associated with follicular maturational competence acquisition in rainbow trout (Oncorhynchus mykiss). Fish Physiol Biochem.

[11] Bobe J, Montfort J, Nguyen T, Fostier A (2006). Identification of new participants in the rainbow trout (Oncorhynchus mykiss) oocyte maturation and ovulation processes using cDNA microarrays. Reprod Biol Endocrinol.

[12] Patiño R, Thomas P, Yoshizaki G (2003). Ovarian follicle maturation and ovulation: an integrated perspective. Fish Physiol Biochem.

[13] Lubzens E, Young G, Bobe J, Cerda J (2010). Oogenesis in teleosts: how eggs are formed. Gen Comp Endocrinol.

[14] Knight OM, Van Der Kraak G (2015). The role of eicosanoids in 17alpha, 20beta-dihydroxy-4-pregnen-3-one-induced ovulation and spawning in Danio rerio. Gen Comp Endocrinol.

[15] Westerfield M (1995). The Zebrafish Book: a Guide for the laboratory Use of Zebrafish (Danio rerio).

[16] Rozen S, Skaletsky H (2000). Primer3 on the WWW for general users and for biologist programmers. Methods Mol Biol.

[17] Goetz FW, Garczynski M (1997). The ovarian regulation of ovulation in teleost fish. Fish Physiol Biochem.

[18] Ogiwara K, Takano N, Shinohara M, Murakami M, Takahashi T (2005). Gelatinase A and membrane-type matrix metalloproteinases 1 and 2 are responsible for follicle rupture during ovulation in the medaka. Proc Natl Acad Sci U S A.

[19] Bobe J, Nguyen T, Fostier A (2009). Ovarian function of the trout preovulatory ovary: new insights from recent gene expression studies. Comp Biochem Physiol A Mol Integr Physiol.

[20] Lin B, Annabi B, Hiraiwa H, Pan CJ, Chou JY (1998). Cloning and characterization of cDNAs encoding a candidate glycogen storage disease type 1b protein in rodents. J Biol Chem.

[21] Lin B, Pan CJ, Chou JY (2000). Human variant glucose-6-phosphate transporter is active in microsomal transport. Hum Genet.

[22] He L, Vasiliou K, Nebert DW (2009). Analysis and update of the human solute carrier (SLC) gene superfamily. Hum Genomics.

[23] Pan CJ, Chen SY, Lee S, Chou JY (2009). Structure-function study of the glucose-6-phosphate transporter, an eukaryotic antiporter deficient in glycogen storage disease type Ib. Mol Genet Metab.

[24] Chen SY, Pan CJ, Nandigama K, Mansfield BC, Ambudkar SV, Chou JY (2008). The glucose-6-phosphate transporter is a phosphate-linked antiporter deficient in glycogen storage disease type Ib and Ic. Faseb J.

[25] Hiraiwa H, Pan CJ, Lin B, Moses SW, Chou JY (1999). Inactivation of the glucose 6-phosphate transporter causes glycogen storage disease type 1b. J Biol Chem.

[26] Kundu BK, Zhong M, Sen S, Davogustto G, Keller SR, Taegtmeyer H (2015). Remodeling of glucose metabolism precedes pressure overload-induced left ventricular hypertrophy: review of a hypothesis. Cardiology.

[27] Tabas I, Ron D (2011). Integrating the mechanisms of apoptosis induced by endoplasmic reticulum stress. Nat Cell Biol.

[28] Kaji K, Oda S, Shikano T, Ohnuki T, Uematsu Y, Sakagami J, Tada N, Miyazaki S, Kudo A (2000). The gamete fusion process is defective in eggs of Cd9-deficient mice. Nat Genet.

[29] Filen S, Lahesmaa R (2010). GIMAP Proteins in T-Lymphocytes. J Signal Transduct.

[30] Krucken J, Schroetel RM, Muller IU, Saidani N, Marinovski P, Benten WP, Stamm O, Wunderlich F (2004). Comparative analysis of the human gimap gene cluster encoding a novel GTPase family. Gene.

[31] Carter C, Dion C, Schnell S, Coadwell WJ, Graham M, Hepburn L, Morgan G, Hutchings A, Pascall JC, Jacobs H (2007). A natural hypomorphic variant of the apoptosis regulator Gimap4/IAN1. J Immunol.

[32] Chen Y, Yu M, Dai X, Zogg M, Wen R, Weiler H, Wang D (2011). Critical role for Gimap5 in the survival of mouse hematopoietic stem and progenitor cells. J Exp Med.

[33] Crespo D, Goetz FW, Planas JV (2015). Luteinizing hormone induces ovulation via tumor necrosis factor alpha-dependent increases in prostaglandin F2alpha in a nonmammalian vertebrate. Sci Rep.

[34] Crespo D, Bonnet E, Roher N, MacKenzie SA, Krasnov A, Goetz FW, Bobe J, Planas JV (2010). Cellular and molecular evidence for a role of tumor necrosis factor alpha in the ovulatory mechanism of trout. Reprod Biol Endocrinol.

[35] Murdoch WJ, McDonnel AC (2002). Roles of the ovarian surface epithelium in ovulation and carcinogenesis. Reproduction.

[36] Gunnarsson L, Kristiansson E, Forlin L, Nerman O, Larsson DG (2007). Sensitive and robust gene expression changes in fish exposed to estrogen--a microarray approach. BMC Genomics.

